# National incidence of traumatic spinal fractures in China: Data from China National Fracture Study

**DOI:** 10.1097/MD.0000000000012190

**Published:** 2018-08-21

**Authors:** Bo Liu, Yanbin Zhu, Song Liu, Wei Chen, Fei Zhang, Yingze Zhang

**Affiliations:** a Department of Orthopaedic Surgery, The Third Hospital of Hebei Medical University; b Key Laboratory of Biomechanics of Hebei Province, Shijiazhuang, Hebei, PR China; c Chinese Academy of Engineering, Beijing, PR China.

**Keywords:** epidemiology, incidence, trauma, traumatic spinal fractures

## Abstract

To provide a basis for disease prevention, this article aimed to clarify the incidence and risk factors of traumatic fractures in China.

The China National Fracture Study (CNFS) was a retrospective epidemiological study that recruited a nationally representative sample from 8 provinces, 24 urban cities, and 24 rural counties in China using stratified random sampling and the probability proportional to size method. A total of 512,187 individuals were involved in CNFS. Incidence rates for traumatic spinal fractures were estimated from the database of CNFS. The distributions by age and sex; as well as by demographic factors such as ethnic origin, occupation, and geographical region were also analyzed. The potential risk factors for traumatic spinal fractures were also identified, such as gender, age, ethnic origin, education, occupation, cigarette smoking, alcohol drinking, calcium or vitamin D taking, body mass index (BMI), sleep time per day, history of previous fracture, and urbanization. This study is registered with the Chinese Clinical Trial Registry, number ChiCTR-EPR-15005878.

Around 168 individuals (92 men and 76 women, mean age 55.36 ± 16.19 years) reported 178 (10 individuals with 2 segments) segments of spinal fractures that had occurred in 2014. The incidence rate for traumatic spinal fractures was 32.80 per 100,000 people. Stratified by occupation, retired and unemployed individuals had the highest incidence rates: 72.45 (42.19–102.71) and 64.08 (36.68–91.48) per 100,000 people. According to education level, illiterate individuals had the highest incidence rate (73.39, 54.00–92.79 per 100,000 population). Among all age groups, fractures of thoracolumbar vertebra (T11-L2) were the most common for both sexes, followed by fractures of lumbar vertebra (L3–L5). Four independent risk factors for traumatic spinal fractures were found, including aging, alcohol drinking, sleeping <7 hours per day, and having a previous fracture history.

The current study provides detailed information about the national incidence of traumatic spinal fractures, distribution, and risk factors. Aging, alcohol drinking, sleeping <7 hours per day, and having a previous fracture history were the independent risk factors for traumatic spinal fractures in China.

## 1 Introduction

Traumatic spinal injuries are one of the most common causes of major morbidity and mortality.^[1]^ Spinal and spine-related injuries, which are common in trauma, have the poorest functional outcomes and the lowest rates of return to work among all major organ injuries.^[2]^ One of the first steps in preventing this injury is the collection and analysis of data to help define the problem and identify possible risk factors in various populations. Several articles concerning the epidemiological features of spinal fractures have been published.^[3–5]^ Hu et al^[6]^ reported that the incidence rate of traumatic spinal fractures was 64 per 100,000 in Manitoba province, Canada. Cooper et al^[7]^ estimated the incidence rate in Rochester, Minnesota which was 117 per 100,000. Grivna et al^[4]^ reported that most patients of spinal injuries were young and mid-aged males in United Arab Emirates. The predominant injury mechanism was traffic accident.^[4]^ While in Chongqing (a city in the west region of China) Wang et al^[8]^ found that 65.5% traumatic spinal fracture patients were males, the peak frequency of these injuries was in 31- to 40-years. Accidental falls were the most common cause^[8]^ (58.9%).

However, these reports were only limited to one or few regions. In addition, most of them were based on patients from one or more trauma centers and focused on the demographic characteristic and trauma mechanism. There is a lack of a population-based epidemiology study on spinal fractures. With a population in excess of 1.36 billion, China is a vast country with substantial diversity in terms of economic development, cultural practices, and health-care systems.^[9]^ Some Chinese studies have reported the epidemiology of spinal fractures, but most studies have been limited by small sample size and restricted geographic areas. The incidence and risk factors for traumatic spinal fractures in the entire Chinese population have not been elucidated. Therefore, we had designed the China National Fracture Study (CNFS) involving 512,187 individuals from various regions, ethnic origins, and occupations throughout China. In the current study, we focused on traumatic spine fractures and their population-based incidence, injury mechanism, place of fracture occurrence and identified some associated risk factors, which was of more pertinence in knowledge and prevention of these injuries.

## 2 Materials and methods

The detailed methods of CNFS had been described previously.^[9]^ The CNFS was a retrospective epidemiological study that recruited a nationally representative sample from 8 provinces, 24 urban cities, and 24 rural counties in China using stratified random sampling and the probability proportional to size method. A total of 512,187 individuals were involved in CNFS. It is registered with the Chinese Clinical Trial Registry, number ChiCTR-EPR-15005878. In this study, all data were available from the database of CNFS. The CNFS was briefly described as follows.

### 2.1 Sampling method and sample size

Probability proportionate to size (PPS) sampling method was used to complete the sampling process. We have initially estimated 510,000 individuals to meet recommended requirements for precision in complex survey design.^[10]^ As China is a vast country with substantial diversity in terms of socioeconomic development, terrain, climate and cultural practices, we have categorized all of the 31 provinces of China into 3 regions: east, central, and west. Initially, 8 provinces were selected from the 3 regions, including 3 provinces from the east region, 2 provinces from the central region and 3 provinces from the west region. Because of the real difference in sociology and economic between city and countryside, for each selected province the sampling was done separately in urban and rural areas.

For urban areas, we have divided cities into large, mid-sized, and small according to the population size. In all sampled provinces, a city was selected in each urban stratum based on the geographical location from west to east on the electronic map by PPS method. Next, we had selected a certain number (1–6) of streets from each selected city and a certain number (1–10) of neighborhood communities from each street. Then the number of families in each community was determined by the average number of household members according to the latest official census data. According to their building, apartment, and room numbers, the families were also selected by PPS method until the required sample size was reached. All family members available were invited to participate in the study except who had been living in their current residence <6 months. For rural areas, similar sampling methods were used until required sample size was reached.

In the urban areas, 24 cities, 112 neighborhood communities were sampled. In the rural areas, 24 counties and 223 administrative villages were sampled. During the sampling phase, 10 communities and 1 village refused to participate, and an additional 8 communities and 23 villages contained fewer individuals than expected. Therefore, these groups were replaced by the resampled neighborhood communities and administrative villages. Initially, 535,836 individuals were selected and invited to participate in the CNFS, but questionnaires from 23,649 (4%) individuals were ultimately excluded due to missing items, insufficient responses, or logical errors. Finally, 512,187 (96%) individuals participated in the CNFS: 259,649 (51%) boys and men and 252,538 (49%) girls and women.

### 2.2 Participants and survey

All eligible family members were personally interviewed by a trained investigator. Only individuals in family were investigated. Individuals who lived in collective dwelling, such as bead house, barracks, boarding schools, and welfare homes were excluded from the CNFS. For insurance of data accuracy, for children <18 years, we had obtained the information from their guardians. If a selected family, neighborhood community, or administrative village was unavailable due to some reasons (e.g., house-moving or refusing to participate), we had chosen another household randomly from the list using a modified version of the Kish method.^[11,12]^

A standardized questionnaire was used to collect the information from the individuals. The questionnaires were made out by trained investigators. This collected information including age, gender, ethnic nationality, marital status, education, smoking, alcohol drinking, occupation, residence, etc. If an individual suffered from a traumatic spinal fracture between January 1, and December 31, 2014, an additional questionnaire was carried out to collect the detailed information (including the date and location of fracture, segment involved, injury mechanism, etc.) of the fracture. The injury mechanisms in this study were categorized as follows: traffic accidents; slips, trips, or falls; falls from heights; crushing injury; sharp trauma; blunt force trauma; and others. To ensure the authenticity and accuracy of the survey, individuals were asked to provide their medical records, radiographs, diagnostic reports, and medical reports. If all of these evidences above were unavailable, the individuals were then taken to the nearest hospital to obtain a new radiograph of the fracture site. The cost of the examination would be paid from the investigators. Only individuals with traumatic fractures which confirmed by evidences listed above were involved in the CNFS. Pathological fractures, fragility fractures, fatigue fractures, and insufficiency fractures were excluded in the study. The CNFS was approved by the Institutional Review Board of the Third Hospital of Hebei Medical University, and all methods were performed in accordance with the Declaration of Helsinki. Written informed consent was obtained from all participants before data collection.

### 2.3 Statistical analysis

All data were collected and a database was built for statistical analysis. Incidence rates for traumatic spinal fractures were estimated for the overall population and for subgroups by age and sex; as well as by demographic factors such as ethnic origin, occupation, geographical region and education level. Differences in incidence between categories of nominal variables, such as occupation, regions and ethnic origin, were tested using the χ^2^ test. Trends in incidence rates by age and education were tested by trend χ^2^ test. A multiple logistic regression model was built to explore the potential risk factors for traumatic spinal fractures. These factors were as follows: gender, age, ethnic origin, education, occupation, cigarette smoking, alcohol drinking, calcium or vitamin D taking, BMI, sleep time per day, history of previous fracture, and urbanization. Odds ratio (OR) and 95% confidence interval (95% CI) were calculated to indicate the strength of correlation of risk factors. All statistical analyses were done with SPSS version 19.0 (SPSS Inc., Chicago, IL). A *P* value <.05 was considered as statistical significance.

## 3 Results

A total of 168 individuals (92 men and 76 women, mean age 55.36 ± 16.19 years) reported 178 (10 individuals with 2 segments) segments of spinal fractures that had occurred in 2014. Among them were 3 (1.79%) children, 115 (68.45%) young and middle-aged adults and 50 (29.76%) older individuals. The incidence rate of traumatic spinal fractures in China was 32.80 (95% CI 27.84–37.76) per 100,000 person-year (Table 1).

**Table 1:**
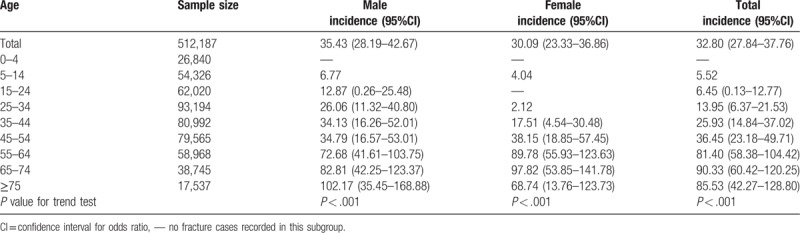
National incidence (incidence rate per 100,000 person-year) of spinal fractures in China by demographic factors.

We also analyzed the incidences of traumatic spinal fracture by individual characteristics and regions. There was no significant difference in incidence between those of Han ethnicity and all other ethnicities combined, nor was there any significant difference according to urbanization. Males in the west region had the highest incidence rate, followed by the east region and the central region. But no differences were found in females between geographic regions. Stratified by occupation, retired and unemployed individuals had the highest incidence rates: 72.45 (42.19–102.71) and 64.08 (36.68–91.48) per 100,000 person-year, respectively. According to education level, illiterate individuals had the highest incidence rate (73.39, 54.00–92.79 per 100,000 person-year) (Table 2).

**Table 2:**
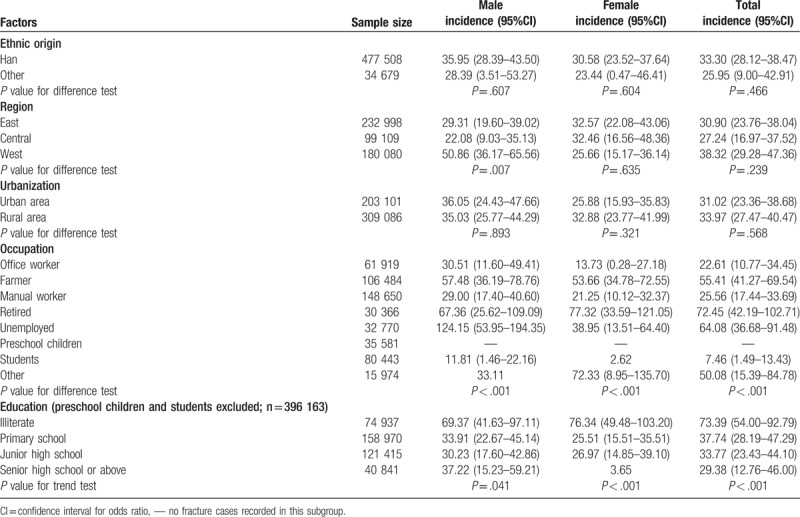
National incidence (incidence rate per 100,000 person-year) of spinal fractures in China by socioeconomic and geographic factors.

The fracture segments were also analyzed. Among all age groups, fractures of thoracolumbar vertebra (T11-L2) were the most common for both sexes (males 15–64 years: 25.10, 17.92–32.27 per 100,000 person-year; females 15–64 years:18.14, 12.04–24.23 per 100,000 person-year; males ≥65 years: 56.88, 29.02–84.74 per 100,000 person-year, females ≥65 years: 60.39, 31.69–89.08 per 100,000 person-year), followed by fractures of lumbar vertebra (L3-L5) (Table 3).

**Table 3:**
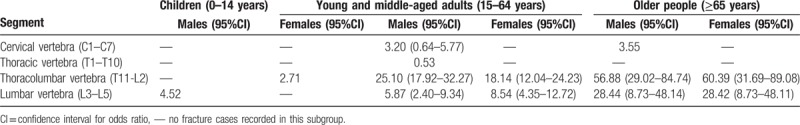
National incidence (incidence rate per 100,000 person-year) of spinal fractures in China by segment in 2014.

We also summarized the proportion of each category of causal mechanisms for young and middle-aged adults and older people. In all subpopulations, injuries occurred most commonly via slips, trips, or falls. Fall from heights was the second most common causes of injury in young and middle-aged males. Fractures caused by traffic accidents accounted for about 1/3 fractures in young and middle-aged males but less than about 1/10 of those in young and middle-aged females. In older population, slip, trip, or fall was the majority cause in both sexes (Table 4).

**Table 4:**
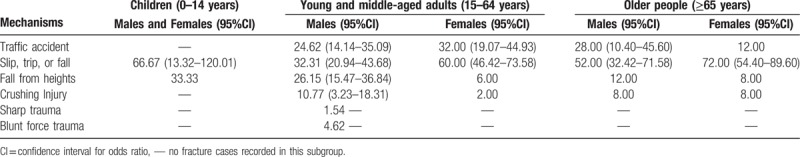
Proportion of spinal fractures by causal mechanisms in China in 2014 (% of total).

Four independent risk factors for traumatic spinal fractures were found. Aging was the strongest risk factor among others. Compared with people aged 15 to 24 years, those aged over 35 years were more likely to experience fractures. In addition, the ORs were continuously increasing along with aging. Alcohol drinking (OR = 1.84, 95%CI = 1.35–2.51), sleeping < 7 hours per day (OR = 1.89, 95%CI = 1.37–2.62), and having a previous fracture history (OR = 3.31, 95%CI = 2.00–5.49) were also identified as risk factors of traumatic spinal fractures (Table 5).

**Table 5:**
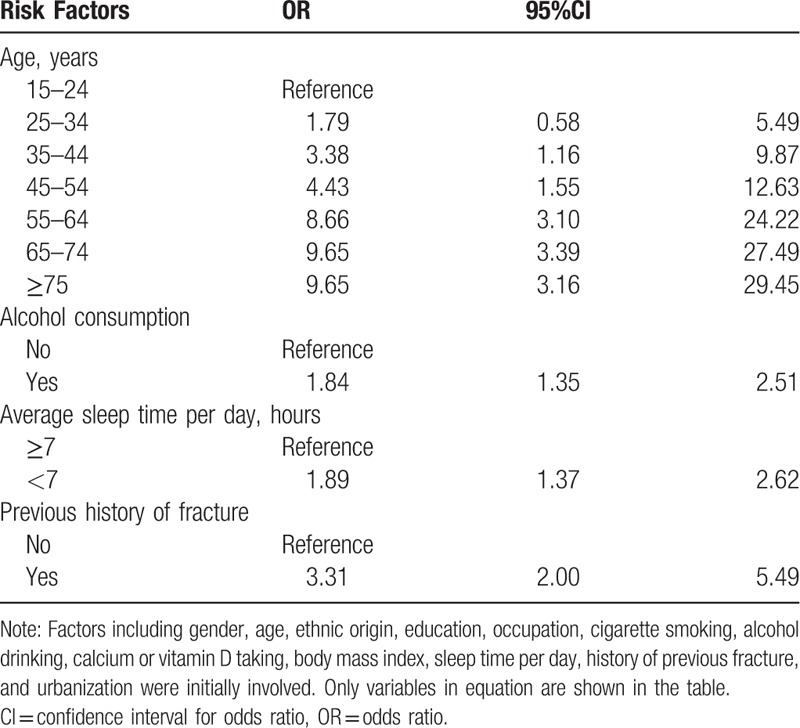
Risk factors for spinal fractures.

## 4 Discussion

The incidence rate for traumatic spinal fractures was 32.80 per 100,000 person-year in 2014, which, by extension, suggests that roughly 450,000 Chinese had a traumatic spinal fracture.^[9]^ The incidence rate was increasing along with aging for both sexes. Additionally, aging was the strongest risk factor in this study. Compared with those who were aged 15 to 24 years, individuals over 35 years had a significant increased risk of traumatic spinal fractures. The odds for fracture risk approximately increased by 3-fold to 4-fold for individuals aged 35 to 54 years and by 8-fold to 9-fold for individuals over 55 years.Melton^[13]^ had found that the age-adjust prevalence of spinal fractures was 6.5% in 50-to 59-year-old age group and 46.5% in the 80- to 89-year-old. Hu et al^[6]^ found an increasing number of spinal fractures with increasing age. Ghaffarifam et al,^[14]^ Tafida et al,^[15]^ and Holmes et al^[16]^ also drawn conclusions similar to our study, which suggested that elderly people were more likely to suffer from spinal fractures. A general aging of the Chinese population has been recorded in recent years, with the proportion of people aged 65 years or older increasing from 8.9% in 2010 to 10.1% in 2014.^[9]^ Therefore, our finding suggested that the incidence of traumatic spinal fractures might increase in the next few years.

Furthermore, osteoporotic fractures in elderly are well known to increase with age, and it is not surprising that the incidence and prevalence of spinal injuries increase markedly with the advance of age.^[17]^ With advancing age, bone mineral density decreases, and a threshold is reached when normal physiologic stresses are no longer safely born.^[18]^ The spinal fractures in elderly might be fragility fractures which were closely related to osteoporosis. In the current study, low energy trauma was the most common cause of this kind of fractures. Thus, prevention and treatment of osteoporosis (such as calcium and vitamin D supplement, bisphosphonates) needs to be emphasized to help reduce the risk of traumatic spinal fractures in elderly.^[19]^ For individuals over 15 years, slips, trips, or falls were the predominant cause. Especially in elderly population, more than half of the traumatic spinal fractures were caused by slips, trips, or falls. Ghaffarifam et al^[14]^ found that the likelihood of all kinds of fall-related injuries increases with age, and older adults are 5 times more likely to be hospitalized due to falls than other injuries. According to their study, fracture of lumbar spine was one of the most common and frequent kinds of results by fall injuries. Tafida et al^[15]^ also reported that the top cause of traumatic spinal fractures as due largely to falls, especially in lumbo-sacral level. These indicated that the prevention of falls and other low energy trauma, especially in the elderly, may reduce the incidence of spinal fractures dramatically.^[1]^ Elderly might benefit from a directed assessment and modification of environmental hazards (e.g., problems with building design, handrails, surfaces, changes in elevation and lighting) to reduce the incidence of falls and traumatic spinal fractures.^[20]^ In addition, by encouraging scientific, reasonable and effective sports therapy which can be used as an effective method to prevent both osteoporosis and falls in elderly,^[21]^ might be another method to decrease the incidence rate of traumatic spinal fracture.

Stratified by occupation, compared with unemployed individuals, males who held a job had a reduced traumatic spinal fracture risk. For females, farmers and retired individuals had a higher incidence for traumatic spinal fracture. Similar to our study, Hu et al^[6]^ found that a higher incidence rate of traumatic spinal fractures in lower income levels, and this might represent differences in lifestyle or occupational exposure to injuring mechanisms. Our results also showed that individuals with a high level of education trends to have a relatively low incidence of spinal fractures. The importance of securing stable employment combined with improvement of education level must therefore become a central component of spinal fracture preventions.

Alcohol consumption was a certain risk factor in this study. Compared to those who do not drink, individuals with alcohol consumption had an increased traumatic spinal fracture risk by 80% in this study. Relative research had shown that alcohol could change bone metabolic, resulting in an osteoporosis and bone strength reduction.^[22]^ Alcohol-related falls were also a potential cause for traumatic spinal fractures.^[14]^ Another risk factor for traumatic spinal fracture in current study was sleeping for <7 hours per day. This is consistent with several previous reports.^[9,13]^ Sleep impairment had already been a certain risk factor for increased injury risk. Therefore, improvement of sleep quality and increasing in the sleep duration might be helpful to reduce the risk of traumatic spinal fractures.

Apart from aging, previous history of fracture was the second strongest risk factor for traumatic spinal fractures in the current study. Similar to our study, Cauley et al^[23]^ found that a previous history of osteoporosis fracture referred to a high incidence rate of hip fractures. Saeidiborojeni and Fattahian^[24]^ also found that previous history of fracture was a risk factor for another fracture in Iran. Duan et al^[18]^ and Liu et al^[25]^ drawn a conclusion that vertebral fractures were often associated with osteoporosis and a history of fractures. Thus, our results suggested that the prevention of secondary fractures should be strengthened and recommended among individuals, especially those with a previous fracture history. Implementation of fall prevention measures and home and behavioral modifications will also be helpful to reduce the risk of secondary fractures.

We have some limitations that should be considered in this study. First, there might have been some selection bias affecting the results. For example, if a patient suffered from a cervical fracture combined with a spinal cord injury and dead following the injury, we could hardly access the information about this individual. Second, recalled bias might exist as the recent fractures might be easily and accurately memorized, but a fracture happening long ago might be forgotten. Third, we only character the fracture segment of spine, but not recorded the position and classification of fractures (e.g. we do not distinguish a vertebral fracture from spinal appendix fracture). Another limitation was that some other information of patients, such as the patient's socioeconomic status, average length of in-hospital stay and hospitals’ levels which may also influence the rates of traumatic spinal fractures, were unavailable in this study. Finally, as an epidemiological study on traumatic spinal fractures, the incidence of spinal cord injury and the treatment and prognosis of patients were still unknown in this study.

## 5 Conclusion

The current study provides detailed information about the national incidence of traumatic spinal fractures, distribution, and risk factors. Elderly, unemployed, and illiterate individuals have a relatively higher incidence of traumatic spinal fractures. Aging, alcohol consumption, sleep <7 hours per day and previous history of fracture are independent risk factors of traumatic spinal fractures. Therefore, specific public health policies which focus on decreasing alcohol consumption and encourage individuals to obtain sufficient sleep should be urgently implemented to help reduce the risk of traumatic spinal fractures. Education and interventions for the prevention of falls and other trauma also need to be emphasized, especially in the elderly and those with a previous fracture history.

## Author contributions

**Conceptualization:** Yanbin Zhu, Yingze Zhang.

**Data curation:** Bo Liu.

**Formal analysis:** Yanbin Zhu, Song Liu, Yingze Zhang.

**Funding acquisition:** Song Liu, Yingze Zhang.

**Investigation:** Song Liu, Fei Zhang, Yingze Zhang.

**Methodology:** Bo Liu, Yanbin Zhu, Song Liu, Wei Chen, Fei Zhang, Yingze Zhang.

**Project administration:** Bo Liu, Song Liu, Wei Chen.

**Resources:** Bo Liu.

**Software:** Yanbin Zhu, Fei Zhang.

**Supervision:** Wei Chen.

**Writing – original draft:** Bo Liu, Yanbin Zhu, Wei Chen, Yingze Zhang.

**Writing – review & editing:** Bo Liu, Yingze Zhang.
